# Re-annotation and re-analysis of the *Campylobacter jejuni *NCTC11168 genome sequence

**DOI:** 10.1186/1471-2164-8-162

**Published:** 2007-06-12

**Authors:** Ozan Gundogdu, Stephen D Bentley, Matt T Holden, Julian Parkhill, Nick Dorrell, Brendan W Wren

**Affiliations:** 1 Pathogen Molecular Department, London School of Hygiene & Tropical Medicine, Keppel Street, UK; 2 Pathogen Sequencing Unit, Sanger Institute, UK

## Abstract

**Background:**

*Campylobacter jejuni *is the leading bacterial cause of human gastroenteritis in the developed world. To improve our understanding of this important human pathogen, the *C. jejuni *NCTC11168 genome was sequenced and published in 2000. The original annotation was a milestone in *Campylobacter *research, but is outdated. We now describe the complete re-annotation and re-analysis of the *C. jejuni *NCTC11168 genome using current database information, novel tools and annotation techniques not used during the original annotation.

**Results:**

Re-annotation was carried out using sequence database searches such as FASTA, along with programs such as TMHMM for additional support. The re-annotation also utilises sequence data from additional *Campylobacter *strains and species not available during the original annotation. Re-annotation was accompanied by a full literature search that was incorporated into the updated EMBL file [EMBL: AL111168]. The *C. jejuni *NCTC11168 re-annotation reduced the total number of coding sequences from 1654 to 1643, of which 90.0% have additional information regarding the identification of new motifs and/or relevant literature. Re-annotation has led to 18.2% of coding sequence product functions being revised.

**Conclusions:**

Major updates were made to genes involved in the biosynthesis of important surface structures such as lipooligosaccharide, capsule and both *O*- and *N*-linked glycosylation. This re-annotation will be a key resource for *Campylobacter *research and will also provide a prototype for the re-annotation and re-interpretation of other bacterial genomes.

## Background

*Campylobacter jejuni *is the leading bacterial cause of human gastroenteritis in the developed world [1]. *C. jejuni *infection has also been associated with post-infection sequelae including septicaemia and neuropathies such as Guillain-Barré Syndrome (GBS) [2]. Infection has largely been linked with the consumption of contaminated poultry or meat products. Given the socioeconomic importance of this pathogen, it is surprising that the ecology, the epidemiology and, in particular, the pathogenesis are still so poorly understood [3]. The lack of information on this problematic pathogen was one of the main driving forces for the original *C. jejuni *NCTC11168 genome project published in 2000 [4], and equally is why a re-annotation and re-analysis of the genome is required.

Since the publication of the *C. jejuni *NCTC11168 genome sequence in 2000, there has been a spectacular increase in research on this important human pathogen. One result of this has been significant revisions of the genetic loci that code for important surface structures on *C. jejuni *strains. The surface polysaccharide region has since been identified as a capsule locus (*Cj1413c *– *Cj1448c*) [5-7]. The flagellar modification locus has been identified as an *O*-linked glycosylation pathway (*Cj1293 *– *Cj1342*) [8-11]. Progress has also been made in our understanding of the lipooligosaccharide (LOS) locus. In addition, the *N*-linked glycosylation pathway has been identified in *C. jejuni *(*Cj1119 *– *Cj1130*) [9,12-14]. This *N*-linked general glycosylation system was initially thought to only be present in eukaryotes. To date, up to 30 proteins modified with the same heptasaccharide glycan structure have been identified. Research over the last 7 years on *C. jejuni*, coupled with the publication of a further 2 *C. jejuni *genome sequences [15,16] and another 3 *Campylobacter *species [15], has heightened the need for re-analysis of the original NCTC11168 genome sequence.

Re-annotation is defined as the process of annotating a previously annotated genome [17]. Examples of re-annotated genomes are unfortunately rare compared to the number of sequenced genomes [18,19]. Clearly the ever-increasing number of new genome sequences requires prioritisation from annotators. Automated methods can save time and resources, but will not incorporate the maximum information available from expert curators, leading to incomplete or even false designations. By contrast, manual annotation is costly and time consuming. However, manual re-annotation of genomes can significantly reduce the perpetuation of errors and thus reduce the time spent on flawed research. Outdated annotations can lead to significant gaps in our knowledge. Hence, there is a need for a research community-wide review and regular update of genome interpretations. Here we have shown the importance of genome re-annotation in terms of maintaining and increasing the usefulness of this resource, a number of years after the original genome sequencing project was completed.

In this study, we describe the re-annotation and re-analysis of the *C. jejuni *NCTC11168 genome. Manual re-annotation of all coding sequences (CDSs) was carried out using current annotation techniques. Literature searches, updates to genome structure and additional unique genome searches were carried out to produce the most comprehensive annotation of any *Campylobacter *genome to date. The re-annotation of the *C. jejuni *NCTC11168 genome also represents a useful model for the re-evaluation of other bacterial genomes.

## Results & Discussion

### Gene number adjustment

A complete re-annotation of the *C. jejuni *NCTC11168 genome was performed resulting in the reduction of the total number of CDSs from 1654 to 1643. This reduction was due to the merging of adjacent CDSs or the removal of CDSs. Three CDSs originally designated as pseudogenes were removed as a result of merging with adjacent pseudogenes. CDSs designated as pseudogenes were also updated to reflect the complete amino acid sequence for the encoded protein regardless of expression. Phase-variable CDSs that contained an intersecting homopolymeric region between adjacent CDSs on separate frames were merged. This allowed the complete amino acid sequence for appropriate genes to be obtained regardless of phase. Re-interpretation of phase-variable CDSs resulted in removal of seven CDSs. CDS (*Cj1520*) was removed because of the recently discovered CRISPR structural moieties [20] (See Structural modifications section in Results & Discussion). In total, 11 CDSs were removed from the re-annotated sequence (Table 1). The accurate identification of all CDSs within the genome has implications for downstream applications, such as mutagenesis, microarray design and proteome analysis.

**Table 1:** CDSs removed or merged from *C. jejuni *NCTC11168 re-annotation.

**Gene Number**	**Type/Description**
Cj0290c/Cj0291c/Cj0292c	Pseudogenes
Cj0968/Cj0969	Pseudogenes
	
Cj0031/Cj0032	Phase-variable
Cj0170/Cj0171	Phase-variable
Cj0628/Cj0629	Phase-variable
Cj1144c/Cj1145c	Phase-variable
Cj1325/Cj1326	Phase-variable
Cj1335/Cj1336	Phase-variable
Cj1677/Cj1678	Phase-variable
	
Cj1520	CRISPR region identified

### Functional annotation update

A systematic re-annotation of all CDSs was performed. For the purpose of this re-annotation, all CDSs with additional information have had an 'updated' note qualifier attached. This qualifier contains consistent free-hand descriptions on recently identified motifs, relevant similarity search results and any characterisation work carried out within *Campylobacter *species/strains or any orthologs in similar microorganisms. Additionally, the 'updated' note qualifier also contains reasoning for including 'putative' or not within the product function. Putative designations infer an accepted product function without definitive evidence. For each CDS, a full literature search was performed. In total, 64.5% of CDSs have had one or more literature qualifier added. Interestingly, from all the literature added (2092), 50.5% have been published after the year 2000. Considering there was no literature qualifier in the original annotation, this data illustrates the depth of research that has been carried out since 2000 and further supports the need to make use of this information in a re-annotation. Detailed statistics on genome modifications are given in Table 2. 18.2% of CDSs have had their product functions updated. 60.5% of CDSs with new product function have been designated with a different functional classification. Additional file 1 gives the outline of functional classification used in this annotation. This description was adopted from the Sanger Institute. A different functional classification may still be within the same field as the previous function, or may be in a completely new area. 97.8% of these CDSs with a new product function and a different functional classification were given a completely new type of functional classification. Additional file's 2, 3 and 4 give in-depth data on the change and distribution of CDSs within these functional categories. Importantly, the number of CDSs in the 'Unknown and other' category has been reduced by 122. Also, the number of CDSs in the 'Miscellaneous' category has risen by 77. This is attributed to the fact that a number of CDSs have new information relating to a product function from uncharacterised motifs and thus the CDSs were not placed into a specific category as yet.

**Table 2:** Genome wide statistics from *C. jejuni *NCTC11168 re-annotation.

CDSs with new motifs identified	+44.9%
CDSs with new literature qualifier	+64.5%
CDSs with new updated note qualifier	+90.0%
CDSs with hypothetical designations in product function	-7.5%
CDSs with conserved hypothetical in product function	+3.7%
CDSs with putative designation in product function	+5.9%
CDSs with new gene qualifier	+6.3%
CDSs with new product function	+18.2%
CDSs with new product function and with a new functional classification	+60.5%
CDS with new product function and a new functional classification with a different type of function	+97.8%

Since the original annotation, significant new information has been derived on the genetic loci encoding the four main carbohydrate surface structures. The *C. jejuni N*-linked glycosylation pathway (not described in the original annotation), has been fully characterised [9,12-14]. This re-annotation includes the nomenclature for the *pglA-K *(*p*rotein *gl*ycosylation) genes and has updated all product functions for genes *Cj1119c *– *Cj1130c*. The LOS locus (*Cj1131c *– *Cj1152c*) described in the original annotation was updated to include recent product functions and gene names including *neuA*1, *B*1, *C*1 and *hldDE *[21-24]. The *O*-linked glycosylation loci (*Cj1293 *– *Cj1342*) involved in flagellar glycosylation, has been updated to include *neu*, *pse *and *maf *genes [8-11]. Finally, the capsule locus (*Cj1413c *– *Cj1448c*), has now been updated to include *kps *and *hdd *genes [5-7].

Additional genome-wide updates were also carried out, of which a large proportion entailed adding specificity to existing product function. For example, the identification of a new PFAM or PROSITE motif has allowed the product function to become further specified e.g. putative transport protein modified to putative MFS (Major Facilitator System) transport protein. A complete list of changes throughout the *C. jejuni *NCTC11168 genome is provided in Additional File 5.

### Pseudogene & phase-variable modification

Pseudogene identification is a challenging process where discrepancies exist between pseudogene assignment techniques [25]. Identifiers include detection of Open Reading Frames (ORF) belonging to a single CDS on multiple frames, the presence of one or more stop codon within a CDS, and extra information from the biology of the microorganism. More recently, comparative genomics has been used as a technique for pseudogene assignment [26]. The number of pseudogenes identified in the original annotation of the *C. jejuni *NCTC11168 genome was 20. We carried out a re-analysis on all pseudogenes in the NCTC11168 genome. The majority of revisions we carried out incorporated multiple features created from different coordinates on more than one frame. This process is often complicated with support needed from FASTA and TBLASTX search results. Completion of this re-analysis resulted in modification of 19 out of 20 pseudogenes (Table 3). The final pseudogene number was 19 due to the merging of two adjacent CDSs designated as pseudogenes (*Cj0968/Cj0969*).

**Table 3:** Pseudogenes in *C. jejuni *NCTC11168 with modification.

**Gene Number**	**Product**	**Modification**
Cj0046	pseudogene (putative sodium:sulfate transmembrane transport protein)	Features introduced within CDS
Cj0072c	pseudogene (putative iron-binding protein)	Features introduced within CDS
Cj0223	pseudogene (putative IgA protease family protein)	Features introduced within CDS
Cj0292c	pseudogene (putative glycerol-3-phosphate transporter)	Merging of multiple CDS
Cj0444	pseudogene (putative TonB-denpendent outer membrane receptor)	Features introduced within CDS
Cj0501	pseudogene (ammonium transporter)	Features introduced within CDS
Cj0565	pseudogene (conserved hypothetical protein)	Features introduced within CDS
Cj0654c	pseudogene (putative transmembrane transport protein)	Features introduced within CDS
Cj0676	pseudogene (potassium-transporting ATPase A chain)	Features introduced within CDS
Cj0678	pseudogene (potassium-transporting ATPase C chain)	Features introduced within CDS
Cj0742	pseudogene (putative outer membrane protein)	Features introduced within CDS
Cj0752	pseudogene (IS element transposase)	Features introduced within CDS
Cj0866	pseudogene (arylsulfatase)	Features introduced within CDS
Cj0969	pseudogene (putative periplasmic protein)	Merging of multiple CDS
Cj1064	pseudogene (nitroreductase)	Features introduced within CDS
Cj1389	pseudogene (putative C4-dicarboxylate anaerobic carrier)	Features introduced within CDS
Cj1395	pseudogene (putative MmgE/PrpD family protein)	Unmodified
Cj1470c	pesudogene (type II protein secretion system F protein)	Features introduced within CDS
Cj1528	pseudogene (putative C4-dicarboxylate anaerobic carrier)	Features introduced within CDS

An example of the difficulty and complexity associated with pseudogene designation is observed when viewing the CDSs *Cj0522*, *Cj0523 *and *Cj0524 *within *C. jejuni *NCTC11168. These three CDSs are represented as one whole CDS on a single frame within *C. jejuni *RM1221 (*Cje0628*). The three CDSs are large enough to be represented as individual CDSs and in *C. jejuni *NCTC11168 have been represented on more than one frame. The question can be asked as to whether these CDSs (which are intact in *C. jejuni *RM1221), represent a pseudogene in *C. jejuni *NCTC11168. Given the fact that in *C. jejuni *RM1221 these three CDSs do actually code for a product (Na/Pi-cotransporter, putative), it is more likely that they represent a pseudogene in *C. jejuni *NCTC11168. In this re-annotation, our intention was to carry out a full mark up of existing pseudogenes, however, the potential for a pseudogene has been noted.

The frequency and importance of pseudogene formation in microorganisms has attained added significance in recent years with the emergence of genome reduction theories and enhanced virulence through pathoadaptive mutations [27,28]. Recent studies have suggested that ever increasing non-functional genes are being identified within microorganisms and in particular are more common in genomes of recently evolved pathogens, than in their benign or free-living relatives [25]. The number and type of predicted pseudogenes within *C. jejuni *NCTC11168 and *C. jejuni *RM1221 are compared in Additional File 6. Observing CDS location rather than CDS function was carried out for this comparison. This was to ensure variation in product function naming does not exclude identical pseudogenes, which are represented on the same row. Currently, the *C. jejuni *81–176 genome has not been fully annotated so could not be used in this comparison. This is also the case for *C. coli *RM2228, *C. lari *RM2100 and *C. upsaliensis *3195 which only have an estimation of pseudogene numbers based on a subset of genes [15]. In *C. jejuni *NCTC11168, 63% (12/19) of the pseudogenes are shared with *C. jejuni *RM1221. In contrast to 19 pseudogenes in *C. jejuni *NCTC11168, *C. jejuni *RM1221 contains 47 pseudogenes. Assuming these are genuine pseudogenes this would imply *C. jejuni *NCTC11168 (1980 human isolate, UK) and *C. jejuni *RM1221 (2000 chicken isolate, US), share a core set of ancestral pseudogenes. Even with the variation of isolation dates, source and geographical location, there is substantial conservation of pseudogene type. It is speculative to suggest when and how the additional pseudogenes in *C. jejuni *RM1221 arose, or when and how the *C. jejuni *NCTC11168 genome lost CDSs as pseudogenes since divergence occurred.

The significance of pseudogenes in early genome annotations were frequently ignored, as these were considered as sequencing artefacts. However, given the recent realisation of the importance of pseudogenes in pathoadaptive mutations, a greater significance is placed on their identification [26,29]. An example of this is the re-analysis of *Escherichia coli *K-12, which has predicted an additional 161 from the original single pseudogene identified [28]. The same study also indicated pseudogenes are continually generated, with existing pseudogenes being eliminated over a period of time [28]. Pseudogenes can accumulate in the genomes of some bacterial species, especially those undergoing processes like niche adaptation, host specialisation or weak selection strength [30]. Analysis of further *Campylobacter *strains and species along with additional epsilon proteobacteria species will aid our understanding on this emerging area of interest. Also, greater understanding of pseudogene dynamics and in particular innovative pseudogene identification techniques will yield more information about the actual number and purpose of these entities within microorganisms.

Phase-variable CDSs containing hypervariable regions were also analysed. The initial annotation gave a number of hypervariable sequences found within the *C. jejuni *genomic shotgun sequence [4]. These hypervariable sequences are scattered throughout the genome, however, there is a large cluster within both the *O*-linked glycosylation and capsule loci. Further research on these loci have illustrated the impact of phase-variation on microorganism pathogenicity [11,31,32]. Table 4 shows phase-variable CDSs that have been modified.

**Table 4:** Merged phase-variable CDSs in *C. jejuni *NCTC11168 re-annotation.

**Gene Number**	**Product**	**Effect**
Cj0031/Cj0032	putative type IIS restriction/modification enzyme	Fusion/separation
Cj0170/Cj0171	hypothetical protein Cj0170	Fusion/separation
Cj0628/Cj0629	putative lipoprotein	Fusion/separation
Cj1144/Cj1145	hypothetical protein Cj1144c	Fusion/separation
Cj1325/Cj1326	putative methyltransferase	Fusion/separation
Cj1335/Cj1336	motility accessory factor (function unknown)	Fusion/separation
Cj1677/Cj1678	putative lipoprotein	Fusion/separation

### Structural modifications

As well as CDS updates, novel features were also added to the re-annotation. For example, the incorporation of the recently identified Clustered Regularly Interspaced Short Palindromic Repeats (CRISPR) regions within *Campylobacter *[20,33,34]. CRISPR regions are thought to be mobile elements. In conjunction with this, the three CDSs upstream of the CRISPR repeats were identified as CRISPR associated proteins and this concurs with existing CRISPR structures. To date, there has only been one identified CRISPR region within *C. jejuni *and this has now been incorporated within the genome. As a result, one CDS (*Cj1520*) has been removed. This CDS was previously annotated as having five repeat regions. Thus, the genome now contains a CRISPR repeat region in place of the removed CDS.

Additional genome searches included RFAM database search to discover any non-coding RNAs. This search identified two new non-coding RNA structures. RFAM RF00169, a bacterial signal recognition particle (SRP) RNA, was identified upstream of *Cj0046*. The SRP is a universally conserved ribonucleoprotein involved in the co-translational targeting of proteins to membranes [35,36]. Also, RFAM RF00059 a thiamin pyrophosphate (TPP) riboswitch (THI element) was identified upstream of *Cj0453 *(thiamin biosynthesis protein ThiC). The RFAM motif is a conserved structure (THI element), involved in thiamin-regulation [37].

The final step of the re-annotation process was the incorporation of Gene Ontology (GO) annotation. GO annotation attempts to link three structured, controlled vocabularies (ontologies) that describe gene products in terms of their associated biological processes, cellular components and molecular functions in a species-independent manner [38]. This re-annotation aims to incorporate GO annotation using two automated methods. Upon submission to EBI, the EMBL file will carry a GOA link that lists the GO annotation identified automatically by EBI. A second version of GO was generated by performing a reciprocal FASTA search using RM1221. This was created and submitted to GeneDB. GO annotation is a valuable feature in current annotation techniques that can expedite systems biology approaches to genome analysis.

## Conclusions

In summary, the re-annotation and re-analysis of the *C. jejuni *NCTC11168 genome sequence has led to substantial updates across the entire genome, incorporating a vast amount of research information performed since the original annotation in 2000 and also integrating data from additional *Campylobacter *species and strains. Major updates include noteworthy modifications to the 4 main surface structure loci in the genome, 18.2% of genome product functions being updated and 90.0% of all CDSs now having additional information. The inclusion of literature searches and a GO annotation alongside genome wide structural modifications has resulted in *C. jejuni *NCTC11168 being the most comprehensively annotated *Campylobacter *genome to date.

## Methods

### Sequence searches

Manual re-annotation of all previously annotated *C. jejuni *NCTC11168 CDSs [4] was carried out based on results from BLASTP [39] and FASTA [40] sequence comparisons using non-redundant databases. Re-annotation was based, wherever possible, on characterised proteins or genes [4]. Additional functional data was provided by using the PFAM [41] and PROSITE [42] motif databases. New searches carried out in this re-annotation included running RFAM [43] database and also the programs TMHMM [44] and SIGNALP [45].

### Literature & additional searches

This re-annotation included a complete literature search of all CDS numbers and gene names using PubMed [46], HighWire Press [47], Scirus [48] and Google Scholar [49]. Artemis software release 8 [50] was used during re-annotation. The re-annotated sequence was submitted to the EMBL public database and also to GeneDB [51] and CampyDB [52]. The EMBL file included an 'original' and 'updated' note qualifier, a 'product' qualifier and each CDS represented with a unique 'locus_tag' qualifier. Appropriate 'gene' qualifiers were also present. The GeneDB submission included all the above and extra qualifiers 'colour' and 'literature'. This re-annotation also included for the first time a Gene Ontology (GO) annotation of the NCTC11168 genome sequence. This was created automatically on submission to EMBL and can be accessed via the GOA link. A separate GO annotation was created within GeneDB by carrying out a reciprocal FASTA comparison with *C. jejuni *RM1221 and adopting the GO annotation of orthologous CDSs.

### Re-designation of pseudogenes

Advances in genome annotation techniques that were unavailable during the original annotation have led to updated interpretation of pseudogenes and phase-variable CDSs. Using guidance from TBLASTX search results, we carried out a full re-analysis of all pseudogenes. CDSs designated as pseudogenes have been updated to reflect the complete amino acid sequence for the encoded protein regardless of expression. This has caused differences from the amino acid sequence of the previous annotation. Some pseudogene modifications entailed merging two or more adjacent, in frame CDSs (previously annotated as separate pseudogene CDSs), to create a single pseudogene containing internal stop codons. In other cases, pseudogene features were created with multiple coordinates representing one or more frameshift in the CDS – these had previously only detailed the start and stop coordinates so did not reflect the true position of the non-mutated CDS. In both cases the assignment of coordinates was based on matches to homologues determined through FASTA searches.

### Re-designation of CDSs with an intersecting homopolymeric tract

CDSs containing an intersecting homopolymeric tract were merged to reflect the complete amino acid sequence for appropriate genes regardless of phase. This is analogous to the scenario described above for frameshifted pseudogenes. This modification was carried out for two CDSs with an intersecting homopolymeric tract. The joining of such CDSs was not undertaken in the original annotation.

## Authors' contributions

OG carried out the re-annotation process and drafted the manuscript. SDB assisted with the re-annotation process. MTH assisted with running additional programs used in the re-annotation. JP, ND and BWW participated in the conception and supervised the design of the study. All authors submitted comments on drafts and read and approved the final manuscript.

## Supplementary Material

Additional File 1 *C. jejuni *functional classification (created at Sanger Institute). Click here for file

Additional File 2 Distribution of functional classification before/after re-annotation. Click here for file

Additional File 3 Changes to functional classification categories before and after the re-annotation. Click here for file

Additional File 4 Changes to CDS functions and functional classifications. Click here for file

Additional File 5 CDSs modified in *C. jejuni *NCTC11168 re-annotation. Click here for file

Additional File 6 Pseudogene comparison between *C. jejuni *NCTC11168 and *C. jejuni *RM1221. Click here for file

## References

[B1] Andersen MT, Brondsted L, Pearson BM, Mulholland F, Parker M, Pin C, Wells JM, Ingmer H (2005). Diverse roles for HspR in Campylobacter jejuni revealed by the proteome, transcriptome and phenotypic characterization of an hspR mutant. Microbiology.

[B2] Nachamkin I, Allos BM, Ho T (1998). Campylobacter species and Guillain-Barre syndrome. Clin Microbiol Rev.

[B3] Dorrell N, Mangan JA, Laing KG, Hinds J, Linton D, Al-Ghusein H, Barrell BG, Parkhill J, Stoker NG, Karlyshev AV, Butcher PD, Wren BW (2001). Whole genome comparison of Campylobacter jejuni human isolates using a low-cost microarray reveals extensive genetic diversity. Genome Res.

[B4] Parkhill J, Wren BW, Mungall K, Ketley JM, Churcher C, Basham D, Chillingworth T, Davies RM, Feltwell T, Holroyd S, Jagels K, Karlyshev AV, Moule S, Pallen MJ, Penn CW, Quail MA, Rajandream MA, Rutherford KM, van Vliet AH, Whitehead S, Barrell BG (2000). The genome sequence of the food-borne pathogen Campylobacter jejuni reveals hypervariable sequences. Nature.

[B5] Karlyshev AV, Linton D, Gregson NA, Lastovica AJ, Wren BW (2000). Genetic and biochemical evidence of a Campylobacter jejuni capsular polysaccharide that accounts for Penner serotype specificity. Mol Microbiol.

[B6] Karlyshev AV, Champion OL, Churcher C, Brisson JR, Jarrell HC, Gilbert M, Brochu D, St Michael F, Li J, Wakarchuk WW, Goodhead I, Sanders M, Stevens K, White B, Parkhill J, Wren BW, Szymanski CM (2005). Analysis of Campylobacter jejuni capsular loci reveals multiple mechanisms for the generation of structural diversity and the ability to form complex heptoses. Mol Microbiol.

[B7] Karlyshev AV, McCrossan MV, Wren BW (2001). Demonstration of polysaccharide capsule in Campylobacter jejuni using electron microscopy. Infect Immun.

[B8] Thibault P, Logan SM, Kelly JF, Brisson JR, Ewing CP, Trust TJ, Guerry P (2001). Identification of the carbohydrate moieties and glycosylation motifs in Campylobacter jejuni flagellin. J Biol Chem.

[B9] Szymanski CM, Logan SM, Linton D, Wren BW (2003). Campylobacter--a tale of two protein glycosylation systems. Trends Microbiol.

[B10] Liu F, Tanner ME (2006). PseG of Pseudaminic Acid Biosynthesis: A UDP-SUGAR HYDROLASE AS A MASKED GLYCOSYLTRANSFERASE. J Biol Chem.

[B11] Karlyshev AV, Linton D, Gregson NA, Wren BW (2002). A novel paralogous gene family involved in phase-variable flagella-mediated motility in Campylobacter jejuni. Microbiology.

[B12] Linton D, Dorrell N, Hitchen PG, Amber S, Karlyshev AV, Morris HR, Dell A, Valvano MA, Aebi M, Wren BW (2005). Functional analysis of the Campylobacter jejuni N-linked protein glycosylation pathway. Mol Microbiol.

[B13] Glover KJ, Weerapana E, Imperiali B (2005). In vitro assembly of the undecaprenylpyrophosphate-linked heptasaccharide for prokaryotic N-linked glycosylation. Proc Natl Acad Sci U S A.

[B14] Kelly J, Jarrell H, Millar L, Tessier L, Fiori LM, Lau PC, Allan B, Szymanski CM (2006). Biosynthesis of the N-linked glycan in Campylobacter jejuni and addition onto protein through block transfer. J Bacteriol.

[B15] Fouts DE, Mongodin EF, Mandrell RE, Miller WG, Rasko DA, Ravel J, Brinkac LM, DeBoy RT, Parker CT, Daugherty SC, Dodson RJ, Durkin AS, Madupu R, Sullivan SA, Shetty JU, Ayodeji MA, Shvartsbeyn A, Schatz MC, Badger JH, Fraser CM, Nelson KE (2005). Major structural differences and novel potential virulence mechanisms from the genomes of multiple campylobacter species. PLoS Biol.

[B16] Hofreuter D, Tsai J, Watson RO, Novik V, Altman B, Benitez M, Clark C, Perbost C, Jarvie T, Du L, Galan JE (2006). Unique features of a highly pathogenic Campylobacter jejuni strain. Infect Immun.

[B17] Ouzounis CA, Karp PD (2002). The past, present and future of genome-wide re-annotation. Genome Biol.

[B18] Camus JC, Pryor MJ, Medigue C, Cole ST (2002). Re-annotation of the genome sequence of *Mycobacterium tuberculosis* H37Rv. Microbiology.

[B19] Dandekar T, Huynen M, Regula JT, Ueberle B, Zimmermann CU, Andrade MA, Doerks T, Sanchez-Pulido L, Snel B, Suyama M, Yuan YP, Herrmann R, Bork P (2000). Re-annotating the *Mycoplasma pneumoniae* genome sequence: adding value, function and reading frames. Nucleic Acids Res.

[B20] Schouls LM, Reulen S, Duim B, Wagenaar JA, Willems RJ, Dingle KE, Colles FM, Van Embden JD (2003). Comparative genotyping of Campylobacter jejuni by amplified fragment length polymorphism, multilocus sequence typing, and short repeat sequencing: strain diversity, host range, and recombination. J Clin Microbiol.

[B21] Guerry P, Ewing CP, Hickey TE, Prendergast MM, Moran AP (2000). Sialylation of lipooligosaccharide cores affects immunogenicity and serum resistance of Campylobacter jejuni. Infect Immun.

[B22] Gilbert M, Karwaski MF, Bernatchez S, Young NM, Taboada E, Michniewicz J, Cunningham AM, Wakarchuk WW (2002). The genetic bases for the variation in the lipo-oligosaccharide of the mucosal pathogen, Campylobacter jejuni. Biosynthesis of sialylated ganglioside mimics in the core oligosaccharide. J Biol Chem.

[B23] Linton D, Karlyshev AV, Hitchen PG, Morris HR, Dell A, Gregson NA, Wren BW (2000). Multiple N-acetyl neuraminic acid synthetase (neuB) genes in Campylobacter jejuni: identification and characterization of the gene involved in sialylation of lipo-oligosaccharide. Mol Microbiol.

[B24] sValvano MA, Messner P, Kosma P (2002). Novel pathways for biosynthesis of nucleotide-activated glycero-manno-heptose precursors of bacterial glycoproteins and cell surface polysaccharides. Microbiology.

[B25] Lerat E, Ochman H (2005). Recognizing the pseudogenes in bacterial genomes. Nucleic Acids Res.

[B26] Lerat E, Ochman H (2004). Psi-Phi: exploring the outer limits of bacterial pseudogenes. Genome Res.

[B27] Harrison PM, Gerstein M (2002). Studying genomes through the aeons: protein families, pseudogenes and proteome evolution. J Mol Biol.

[B28] Ochman H, Davalos LM (2006). The nature and dynamics of bacterial genomes. Science.

[B29] Homma K, Fukuchi S, Kawabata T, Ota M, Nishikawa K (2002). A systematic investigation identifies a significant number of probable pseudogenes in the *Escherichia coli* genome. Gene.

[B30] Mira A, Pushker R (2005). The silencing of pseudogenes. Mol Biol Evol.

[B31] Linton D, Karlyshev AV, Wren BW (2001). Deciphering Campylobacter jejuni cell surface interactions from the genome sequence. Curr Opin Microbiol.

[B32] Szymanski CM, Michael FS, Jarrell HC, Li J, Gilbert M, Larocque S, Vinogradov E, Brisson JR (2003). Detection of conserved N-linked glycans and phase-variable lipooligosaccharides and capsules from campylobacter cells by mass spectrometry and high resolution magic angle spinning NMR spectroscopy. J Biol Chem.

[B33] Godde JS, Bickerton A (2006). The repetitive DNA elements called CRISPRs and their associated genes: evidence of horizontal transfer among prokaryotes. J Mol Evol.

[B34] Jansen R, van Embden JD, Gaastra W, Schouls LM (2002). Identification of a novel family of sequence repeats among prokaryotes. Omics.

[B35] Rosenblad MA, Gorodkin J, Knudsen B, Zwieb C, Samuelsson T (2003). SRPDB: Signal Recognition Particle Database. Nucleic Acids Res.

[B36] Regalia M, Rosenblad MA, Samuelsson T (2002). Prediction of signal recognition particle RNA genes. Nucleic Acids Res.

[B37] Rodionov DA, Vitreschak AG, Mironov AA, Gelfand MS (2002). Comparative genomics of thiamin biosynthesis in procaryotes. New genes and regulatory mechanisms. J Biol Chem.

[B38] Ashburner M, Ball CA, Blake JA, Botstein D, Butler H, Cherry JM, Davis AP, Dolinski K, Dwight SS, Eppig JT, Harris MA, Hill DP, Issel-Tarver L, Kasarskis A, Lewis S, Matese JC, Richardson JE, Ringwald M, Rubin GM, Sherlock G (2000). Gene ontology: tool for the unification of biology. The Gene Ontology Consortium. Nat Genet.

[B39] Altschul SF, Gish W, Miller W, Myers EW, Lipman DJ (1990). Basic local alignment search tool. J Mol Biol.

[B40] Pearson WR, Lipman DJ (1988). Improved tools for biological sequence comparison. Proc Natl Acad Sci U S A.

[B41] Sonnhammer EL, Eddy SR, Durbin R (1997). Pfam: a comprehensive database of protein domain families based on seed alignments. Proteins.

[B42] Falquet L, Pagni M, Bucher P, Hulo N, Sigrist CJ, Hofmann K, Bairoch A (2002). The PROSITE database, its status in 2002. Nucleic Acids Res.

[B43] Griffiths-Jones S, Bateman A, Marshall M, Khanna A, Eddy SR (2003). Rfam: an RNA family database. Nucleic Acids Res.

[B44] Sonnhammer EL, von Heijne G, Krogh A (1998). A hidden Markov model for predicting transmembrane helices in protein sequences. Proc Int Conf Intell Syst Mol Biol.

[B45] Nielsen H, Engelbrecht J, Brunak S, von Heijne G (1997). Identification of prokaryotic and eukaryotic signal peptides and prediction of their cleavage sites. Protein Eng.

[B46] Entrez Pubmed NIH http://www.ncbi.nlm.nih.gov/entrez/query.fcgi.

[B47] HighWire Press SU http://highwire.stanford.edu/.

[B48] Scirus - for scientific information only E http://www.scirus.com/srsapp/.

[B49] Scholar G http://scholar.google.com/.

[B50] Rutherford K, Parkhill J, Crook J, Horsnell T, Rice P, Rajandream MA, Barrell B (2000). Artemis: sequence visualization and annotation. Bioinformatics.

[B51] Hertz-Fowler C, Peacock CS, Wood V, Aslett M, Kerhornou A, Mooney P, Tivey A, Berriman M, Hall N, Rutherford K, Parkhill J, Ivens AC, Rajandream MA, Barrell B (2004). GeneDB: a resource for prokaryotic and eukaryotic organisms. Nucleic Acids Res.

[B52] Chaudhuri RR, Khan AM, Pallen MJ (2004). coliBASE: an online database for Escherichia coli, Shigella and Salmonella comparative genomics. Nucleic Acids Res.

