# Supplementary material for: Re-annotation and re-analysis of the Campylobacter jejuni NCTC11168 genome sequence
Source: BMC Genomics. 2007 Jun 12;8:162. doi: 10.1186/1471-2164-8-162 (PMC1899501; doi:10.1186/1471-2164-8-162)
Supplement: Additional File 1 — C. jejuni functional classification (created at Sanger Institute). [file 1471-2164-8-162-S1.doc]

- 1 Small molecule metabolism
  - 1.A Degradation
    - [1.A.1](http://www.sanger.ac.uk/Projects/C_jejuni/functional_classes/1.A.1.shtml) Carbon compounds [3, 5] {+2}
    - [1.A.2](http://www.sanger.ac.uk/Projects/C_jejuni/functional_classes/1.A.2.shtml) Amino acids [3, 3]
  - 1.B Energy metabolism
    - [1.B.1](http://www.sanger.ac.uk/Projects/C_jejuni/functional_classes/1.B.1.shtml) Glycolysis [8, 9] {+1}
    - [1.B.3](http://www.sanger.ac.uk/Projects/C_jejuni/functional_classes/1.B.3.shtml) Tricarboxylic acid cycle [13, 13]
    - 1.B.5 Pentose phosphate pathway
      - [1.B.5.b](http://www.sanger.ac.uk/Projects/C_jejuni/functional_classes/1.B.5.b.shtml) Non-oxidative branch [3, 3]
    - [1.B.7](http://www.sanger.ac.uk/Projects/C_jejuni/functional_classes/1.B.7.shtml) Respiration [11, 11]
      - [1.B.7.a](http://www.sanger.ac.uk/Projects/C_jejuni/functional_classes/1.B.7.a.shtml) Aerobic [14, 14]
      - [1.B.7.b](http://www.sanger.ac.uk/Projects/C_jejuni/functional_classes/1.B.7.b.shtml) Anaerobic [4, 4]
      - [1.B.7.c](http://www.sanger.ac.uk/Projects/C_jejuni/functional_classes/1.B.7.c.shtml) Electron transport [41, 44] {+3}
    - [1.B.9](http://www.sanger.ac.uk/Projects/C_jejuni/functional_classes/1.B.9.shtml) ATP-proton motive force [9, 9]
  - 1.C Central intermediary metabolism
    - [1.C.1](http://www.sanger.ac.uk/Projects/C_jejuni/functional_classes/1.C.1.shtml) General [17, 18] {+1}
    - [1.C.2](http://www.sanger.ac.uk/Projects/C_jejuni/functional_classes/1.C.2.shtml) Gluconeogenesis [5, 5]
    - [1.C.3](http://www.sanger.ac.uk/Projects/C_jejuni/functional_classes/1.C.3.shtml) Sugar nucleotides [2, 2]
    - [1.C.4](http://www.sanger.ac.uk/Projects/C_jejuni/functional_classes/1.C.4.shtml) Amino sugars [1, 1]
    - [1.C.5](http://www.sanger.ac.uk/Projects/C_jejuni/functional_classes/1.C.5.shtml) Sulphur metabolism [4, 4]
  - 1.D Amino acid biosynthesis
    - [1.D.1](http://www.sanger.ac.uk/Projects/C_jejuni/functional_classes/1.D.1.shtml) Glutamate family [10, 10]
    - [1.D.2](http://www.sanger.ac.uk/Projects/C_jejuni/functional_classes/1.D.2.shtml) Aspartate family [17, 17]
    - [1.D.3](http://www.sanger.ac.uk/Projects/C_jejuni/functional_classes/1.D.3.shtml) Serine family [6, 6]
    - [1.D.4](http://www.sanger.ac.uk/Projects/C_jejuni/functional_classes/1.D.4.shtml) Aromatic amino acid family [15, 15]
    - [1.D.5](http://www.sanger.ac.uk/Projects/C_jejuni/functional_classes/1.D.5.shtml) Histidine [8, 8]
    - [1.D.7](http://www.sanger.ac.uk/Projects/C_jejuni/functional_classes/1.D.7.shtml) Branched chain family [10, 10]
  - [1.E](http://www.sanger.ac.uk/Projects/C_jejuni/functional_classes/1.E.shtml) Polyamine synthesis [1, 1]
  - 1.F Purines, pyrimidines, nucleosides and nucleotides
    - [1.F.1](http://www.sanger.ac.uk/Projects/C_jejuni/functional_classes/1.F.1.shtml) Purine ribonucleotide biosynthesis [17, 19] {+2}
    - [1.F.2](http://www.sanger.ac.uk/Projects/C_jejuni/functional_classes/1.F.2.shtml) Pyrimidine ribonucleotide biosynthesis [9, 9]
    - [1.F.3](http://www.sanger.ac.uk/Projects/C_jejuni/functional_classes/1.F.3.shtml) 2'-deoxyribonucleotide biosynthesis [5, 5]
    - [1.F.4](http://www.sanger.ac.uk/Projects/C_jejuni/functional_classes/1.F.4.shtml) Salvage of nucleosides and nucleotides [4, 4]
    - [1.F.5](http://www.sanger.ac.uk/Projects/C_jejuni/functional_classes/1.F.5.shtml) Miscellaneous nucleoside/nucleotide reactions [2, 3] {+1}
  - 1.G Biosynthesis of cofactors, prosthetic groups and carriers
    - [1.G.1](http://www.sanger.ac.uk/Projects/C_jejuni/functional_classes/1.G.1.shtml) Biotin [6, 6]
    - [1.G.10](http://www.sanger.ac.uk/Projects/C_jejuni/functional_classes/1.G.10.shtml) Thioredoxin [2, 2]
    - [1.G.11](http://www.sanger.ac.uk/Projects/C_jejuni/functional_classes/1.G.11.shtml) Menaquinone and ubiquinine [4, 4]
    - [1.G.12](http://www.sanger.ac.uk/Projects/C_jejuni/functional_classes/1.G.12.shtml) Heme [7, 8] {+1}
    - [1.G.2](http://www.sanger.ac.uk/Projects/C_jejuni/functional_classes/1.G.2.shtml) Folic acid [7, 8] {+1}
    - [1.G.4](http://www.sanger.ac.uk/Projects/C_jejuni/functional_classes/1.G.4.shtml) Molybdopterin [8, 9] {+1}
    - [1.G.5](http://www.sanger.ac.uk/Projects/C_jejuni/functional_classes/1.G.5.shtml) Pantothenate [3, 6] {+3}
    - [1.G.6](http://www.sanger.ac.uk/Projects/C_jejuni/functional_classes/1.G.6.shtml) Pyridoxine [2, 4] {+2}
    - [1.G.7](http://www.sanger.ac.uk/Projects/C_jejuni/functional_classes/1.G.7.shtml) Pyridine nucleotide [1, 1]
    - [1.G.8](http://www.sanger.ac.uk/Projects/C_jejuni/functional_classes/1.G.8.shtml) Thiamine [8, 10] {+2}
    - [1.G.9](http://www.sanger.ac.uk/Projects/C_jejuni/functional_classes/1.G.9.shtml) Riboflavin [6, 6]
  - [1.H](http://www.sanger.ac.uk/Projects/C_jejuni/functional_classes/1.H.shtml) Fatty acid biosynthesis [21, 21]
- [2](http://www.sanger.ac.uk/Projects/C_jejuni/functional_classes/2.shtml) Broad regulatory functions [23, 28] {+5}
  - [2.1](http://www.sanger.ac.uk/Projects/C_jejuni/functional_classes/2.1.shtml) Signal transduction [25, 26] {+1}
- 3 Macromolecule metabolism
  - 3.A Synthesis and modification of macromolecules
    - 3.A.1 rRNA and stable RNAs
    - [3.A.11](http://www.sanger.ac.uk/Projects/C_jejuni/functional_classes/3.A.11.shtml) Phospholipids [8, 8]
    - [3.A.2](http://www.sanger.ac.uk/Projects/C_jejuni/functional_classes/3.A.2.shtml) Ribosomal protein synthesis and modification [54, 54]
    - [3.A.3](http://www.sanger.ac.uk/Projects/C_jejuni/functional_classes/3.A.3.shtml) Ribosome maturation and modification [5, 6] {+1}
    - [3.A.5](http://www.sanger.ac.uk/Projects/C_jejuni/functional_classes/3.A.5.shtml) Aminoacyl tRNA synthetases and their modification [36, 40] {+4}
    - [3.A.6](http://www.sanger.ac.uk/Projects/C_jejuni/functional_classes/3.A.6.shtml) Nucleoproteins [1, 1]
    - [3.A.7](http://www.sanger.ac.uk/Projects/C_jejuni/functional_classes/3.A.7.shtml) DNA replication, restriction/modification, recombination and repair [58, 62] {-2} {+6}
    - [3.A.8](http://www.sanger.ac.uk/Projects/C_jejuni/functional_classes/3.A.8.shtml) Protein translation and modification [24, 27] {+3}
    - [3.A.9](http://www.sanger.ac.uk/Projects/C_jejuni/functional_classes/3.A.9.shtml) RNA synthesis, RNA modification and DNA transcription [16, 18] {+2}
  - 3.B Degradation of macromolecules
    - [3.B.2](http://www.sanger.ac.uk/Projects/C_jejuni/functional_classes/3.B.2.shtml) DNA [2, 3] {+1}
    - [3.B.3](http://www.sanger.ac.uk/Projects/C_jejuni/functional_classes/3.B.3.shtml) Proteins, peptides and glycopeptides [15, 23] {+8}
  - [3.C](http://www.sanger.ac.uk/Projects/C_jejuni/functional_classes/3.C.shtml) Cell envelope [1, 1]
    - [3.C.1](http://www.sanger.ac.uk/Projects/C_jejuni/functional_classes/3.C.1.shtml) Membranes, lipoproteins and porins [158, 137] {-26} {+5}
    - [3.C.2](http://www.sanger.ac.uk/Projects/C_jejuni/functional_classes/3.C.2.shtml) Surface polysaccharides, lipopolysaccharides and antigens [71, 70] {-4}{+3}
    - [3.C.3](http://www.sanger.ac.uk/Projects/C_jejuni/functional_classes/3.C.3.shtml) Surface structures [41, 47] {+6}
    - [3.C.4](http://www.sanger.ac.uk/Projects/C_jejuni/functional_classes/3.C.4.shtml) Murein sacculus and peptidoglycan [17, 17]
    - [3.C.5](http://www.sanger.ac.uk/Projects/C_jejuni/functional_classes/3.C.5.shtml) Miscellaneous periplasmic proteins [111, 100] {-12} {+1}
- 4 Cell processes
  - 4.A Transport/binding proteins
    - [4.A.1](http://www.sanger.ac.uk/Projects/C_jejuni/functional_classes/4.A.1.shtml) Amino acids and amines [21, 21]
    - [4.A.2](http://www.sanger.ac.uk/Projects/C_jejuni/functional_classes/4.A.2.shtml) Cations [34, 41] {+7}
    - [4.A.3](http://www.sanger.ac.uk/Projects/C_jejuni/functional_classes/4.A.3.shtml) Carbohydrates, organic acids and alcohols [7, 10] {+3}
    - [4.A.5](http://www.sanger.ac.uk/Projects/C_jejuni/functional_classes/4.A.5.shtml) Anions [10, 10]
    - [4.A.6](http://www.sanger.ac.uk/Projects/C_jejuni/functional_classes/4.A.6.shtml) Other [66, 74] {-3} {+11}
  - [4.B](http://www.sanger.ac.uk/Projects/C_jejuni/functional_classes/4.B.shtml) Chaperones, chaperonins, heat shock [17, 17] {-1) {+1}
  - [4.C](http://www.sanger.ac.uk/Projects/C_jejuni/functional_classes/4.C.shtml) Cell division [8, 9] {+1}
  - [4.D](http://www.sanger.ac.uk/Projects/C_jejuni/functional_classes/4.D.shtml) Chemotaxis and mobility [9, 9]
  - [4.E](http://www.sanger.ac.uk/Projects/C_jejuni/functional_classes/4.E.shtml) Protein and peptide secretion [15, 20] {+5}
  - [4.G](http://www.sanger.ac.uk/Projects/C_jejuni/functional_classes/4.G.shtml) Detoxification [7, 7]
  - [4.I](http://www.sanger.ac.uk/Projects/C_jejuni/functional_classes/4.I.shtml) Pathogenicity [13, 15] {+2}
- 5 Other
  - [5.A](http://www.sanger.ac.uk/Projects/C_jejuni/functional_classes/5.A.shtml) IS elements [1, 1]
  - [5.C](http://www.sanger.ac.uk/Projects/C_jejuni/functional_classes/5.C.shtml) Plasmid related functions [1, 1]
  - [5.D](http://www.sanger.ac.uk/Projects/C_jejuni/functional_classes/5.D.shtml) Drug/analogue sensitivity [8, 9] {+1}
  - [5.F](http://www.sanger.ac.uk/Projects/C_jejuni/functional_classes/5.F.shtml) Adaptions and atypical conditions [2, 2]
  - [5.G](http://www.sanger.ac.uk/Projects/C_jejuni/functional_classes/5.G.shtml) Antibiotic resistance [13, 12] {-1}
  - [5.H](http://www.sanger.ac.uk/Projects/C_jejuni/functional_classes/5.H.shtml) Conserved hypothetical proteins [226, 125] {-104} {+3}
  - [5.I](http://www.sanger.ac.uk/Projects/C_jejuni/functional_classes/5.I.shtml) Unknown [138, 117] {-21}
- 6 Misc
  - [6.A](http://www.sanger.ac.uk/Projects/C_jejuni/functional_classes/6.A.shtml) Misc [75, 152] {-4} {+81}

Additional file 1. *C. jejuni* functional classification (created at Sanger Institute). [] indicates number of CDSs within each category [before re-annotation, after re-annotation]. {} indicates number of CDSs removed or added to each category after re-annotation. The web address is; <http://www.sanger.ac.uk/Projects/C_jejuni/Cj_gene_list_hierarchical.shtml>. A link is provided to CDSs in each category.
