# Supplementary material for: Re-annotation and re-analysis of the Campylobacter jejuni NCTC11168 genome sequence
Source: BMC Genomics. 2007 Jun 12;8:162. doi: 10.1186/1471-2164-8-162 (PMC1899501; doi:10.1186/1471-2164-8-162)
Supplement: Additional File 2 — Distribution of functional classification before/after re-annotation. [file 1471-2164-8-162-S2.doc]

Additional file 2. Distribution of functional classification before/after re-annotation.
