# Supplementary material for: Re-annotation and re-analysis of the Campylobacter jejuni NCTC11168 genome sequence
Source: BMC Genomics. 2007 Jun 12;8:162. doi: 10.1186/1471-2164-8-162 (PMC1899501; doi:10.1186/1471-2164-8-162)
Supplement: Additional File 3 — Changes to functional classification categories before and after the re-annotation. [file 1471-2164-8-162-S3.doc]

| **Number of CDSs before re-annotation** | **Number of CDSs after re-annotation** | **Number of CDSs changed in each category** | **Percentage change (%)** | **Functional Category** |
| --- | --- | --- | --- | --- |
| 317 | 337 | +20 | +6.3 | Small molecule metabolism |
| 48 | 54 | +6 | +12.5 | Broad regulatory functions |
| 618 | 614 | -4 | -0.6 | Synthesis and modification of macromolecules |
| 207 | 233 | +26 | +12.6 | Cell processes |
| 389 | 267 | -122 | -31.4 | Unknown and other |
| 75 | 152 | +77 | +102.7 | Miscellaneous |

Additional file 3. Changes to functional classification categories before and after the re-annotation.
