# Supplementary material for: Re-annotation and re-analysis of the Campylobacter jejuni NCTC11168 genome sequence
Source: BMC Genomics. 2007 Jun 12;8:162. doi: 10.1186/1471-2164-8-162 (PMC1899501; doi:10.1186/1471-2164-8-162)
Supplement: Additional File 5 — CDSs modified in C. jejuni NCTC11168 re-annotation. [file 1471-2164-8-162-S5.doc]

| **Gene Number** | **Previous Function** | **New Function** |
| --- | --- | --- |
|  |  |  |
| Cj0004c | Putative periplasmic protein | monohaem cytochrome C |
| Cj0006 | putative integral membrane protein | putative Na+/H+ antiporter family protein |
| Cj0017c | putative ATP /GTP binding protein | disulphide bond formation protein |
| Cj0018c | small hydrophobic protein | disulphide bond formation protein |
| Cj0021c | hypothetical protein Cj0021c | putative fumarylacetoacetate (FAA) hydrolase family protein |
| Cj0025c | putative transmembrane symporter | putative sodium:dicarboxylate family transmembrane symporter |
| Cj0026c | hypothetical protein Cj0026c | thymidylate synthase |
| Cj0038c | putative membrane protein | putative poly(A) polymerase family protein |
| Cj0041 | hypothetical protein Cj0041 | putative flagellar hook-length control protein |
| Cj0046 | pseudogene (transmembrane transport protein) | pseudogene (putative sodium:sulfate transmembrane transport protein) |
| Cj0054c | hypothetical protein Cj0054c | putative lysine decarboxylase family protein |
| Cj0058 | putative periplasmic protein | putative peptidase C39 family protein |
| Cj0067 | hypothetical protein Cj0067 | putative amidohydrolase family protein |
| Cj0109 | exbB\tolQ family transport protein | putative MotA/TolQ/ExbB proton channel family protein |
| Cj0112 | periplasmic protein | putative TolB precursor protein |
| Cj0115 | peptidyl-prolyl cis-trans isomerase | FKBP-type peptidyl-prolyl cis-trans isomerase |
| Cj0119 | hypothetical protein Cj0119 | putative hydrolase |
| Cj0123c | putative transcriptional regulator | putative tRNA-dihydrouridine synthase |
| Cj0125c | dksA-like protein | hypothetical protein Cj0125c |
| Cj0128c | suhB-like protein | putative inositol monophosphatase family protein |
| Cj0131 | putative periplasmic protein | putative peptidase M23 family protein |
| Cj0133 | hypothetical protein Cj0133 | putative glycoprotease family protein |
| Cj0137 | hypothetical protein Cj0137 | putative ribosome-binding factor A |
| Cj0145 | hypothetical protein Cj0145 | putative TAT (Twin-Arginine Translocation) pathway signal sequence domain protein |
| Cj0154c | putative methylase | putative tetrapyrrole methylase family protein |
| Cj0159c | hypothetical protein Cj0159c | putative 6-pyruvoyl tetrahydropterin synthase |
| Cj0160c | hypothetical protein Cj0160c | putative radical SAM domain protein |
| Cj0172c | hypothetical protein Cj0172c | putative saccharopine dehydrogenase |
| Cj0177 | putative lipoprotein | putative iron transport protein |
| Cj0178 | putative outer membrane siderophore receptor | putative TonB-denpendent outer membrane receptor |
| Cj0186c | putative integral membrane protein | putative TerC family integral membrane protein |
| Cj0188c | hypothetical protein Cj0188c | putative kinase |
| Cj0203 | putative transmembrane transport protein | putative citrate transporter |
| Cj0204 | putative integral membrane protein | putative oligopeptide transporter, OPT family |
| Cj0227 | acetylornithine aminotransferase | putative acetylornithine/succinyldiaminopimelate aminotransferase |
| Cj0230c | hypothetical protein Cj0230c | putative transferase protein |
| Cj0238 | putative integral membrane protein | putative mechanosensitive ion channel family protein |
| Cj0250c | putative transmembrane transport protein | putative MFS (Major Facilitator Superfamily) transport protein |
| Cj0256 | putative integral membrane protein | putative sulfatase family protein |
| Cj0258 | putative helix-turn-helix motif protein | putative bacterial regulatory protein, ArsR family |
| Cj0261c | hypothetical protein Cj0261c | putative SAM-dependent methyltransferase |
| Cj0263 | putative integral membrane protein | zinc transporter |
| Cj0270 | putative tautomerase family protein | putative tautomerase family protein |
| Cj0277 | putative periplasmic protein | homolog of E. coli rod shape-determining protein |
| Cj0292c | pseudogene (partial glpT) | pseudogene (putative glycerol-3-phosphate transporter) |
| Cj0302c | hypothetical protein Cj0302c | putative molybdenum-pterin binding protein |
| Cj0311 | ctc protein homolog | putative 50S ribosomal protein L25 (general stress protein Ctc) |
| Cj0315 | hypothetical protein Cj0315 | putative HAD-superfamily hydrolase, subfamily IIA |
| Cj0327 | hypothetical protein Cj0327 | putative endoribonuclease L-PSP family protein |
| Cj0339 | putative transmembrane transport protein | putative MFS (Major Facilitator Superfamily) transport protein |
| Cj0356c | hypothetical protein Cj0356c | putative dihydroneopterin aldolase |
| Cj0360 | putative phospho-sugar mutase | phosphoglucosamine mutase |
| Cj0363c | putative oxidoreductase | putative oxygen-independent coproporphyrinogen III oxidase |
| Cj0365c | putative outer membrane channel protein | outer membrane channel protein CmeC (multidrug efflux system CmeABC) |
| Cj0367c | putative membrane fusion component of efflux system | periplasmic fusion protein CmeA (multidrug efflux system CmeABC) |
| Cj0372 | hypothetical protein Cj0372 | putative glutathionylspermidine synthase |
| Cj0378c | putative integral membrane protein | putative ferric reductase-like transmembrane protein |
| Cj0379c | hypothetical protein Cj0379c | putative molybdenum containing oxidoreductase |
| Cj0393c | putative oxidoreductase | putative malate:quinone oxidoreductase |
| Cj0394c | hypothetical protein Cj0394c | putative transcriptional activator |
| Cj0399 | putative integral membrane protein | colicin V production protein homolog |
| Cj0415 | putative oxidoreductase subunit | putative GMC oxidoreductase subunit |
| Cj0419 | hypothetical protein Cj0419 | putative histidine triad (HIT) family protein |
| Cj0434 | phosphoglycerate mutase | 2,3-bisphosphoglycerate-independent phosphoglycerate mutase |
| Cj0436 | hypothetical protein Cj0436 | putative pyridoxamine 5'-phosphate oxidase |
| Cj0447 | hypothetical protein Cj0447 | putative NUDIX hydrolase family protein |
| Cj0458c | hypothetical protein Cj0458c | putative tRNA 2-methylthioadenosine synthase |
| Cj0461c | putative integral membrane protein | putative MFS (Major Facilitator Superfamily) transport protein |
| Cj0462 | hypothetical protein Cj0462 | putative radical SAM domain protein |
| Cj0465c | hypothetical protein Cj0465c | group III truncated haemoglobin |
| Cj0481 | putative lyase | putative dihydrodipicolinate synthase |
| Cj0484 | transmembrane transport protein | putative MFS (Major Facilitator Superfamily) transport protein |
| Cj0487 | hypothetical protein Cj0487 | putative amidohydrolase |
| Cj0494 | hypothetical protein Cj0494 | putative exporting protein |
| Cj0495 | hypothetical protein Cj0495 | putative methyltransferase domain protein |
| Cj0499 | HIT-like protein | putative histidine triad (HIT) family protein |
| Cj0500 | putative ATP /GTP binding protein | putative rhodanese-like domain protein |
| Cj0504c | hypothetical protein Cj0504c | putative oxidoreductase |
| Cj0513 | hypothetical protein Cj0513 | phosphoribosylformylglycinamidine (FGAM) synthase |
| Cj0519 | hypothetical protein Cj0519 | putative rhodanese-like domain protein |
| Cj0522 | putative membrane protein | putative Na+/Pi cotransporter protein |
| Cj0529c | hypothetical protein Cj0529c | putative aminodeoxychorismate lyase family protein |
| Cj0540 | hypothetical protein Cj0540 | putative exporting protein |
| Cj0546 | hypothetical protein Cj0546 | putative 3-octaprenyl-4-hydroxybenzoate carboxy-lyase |
| Cj0552 | hydrophobic protein | putative membrane protein |
| Cj0555 | putative integral membrane protein | putative dicarboxylate carrier protein MatC |
| Cj0556 | hypothetical protein Cj0556 | putative amidohydrolase family protein |
| Cj0559 | oxidoreductase | putative pyridine nucleotide-disulphide oxidoreductase |
| Cj0560 | putative integral membrane protein | putative MATE family transport protein |
| Cj0573 | hypothetical protein Cj0573 | putative GatB/Yqey family protein |
| Cj0579c | hypothetical protein Cj0579c | sec-independant protein translocase |
| Cj0580c | putative oxidoreductase | putative oxygen-independent coproporphyrinogen III oxidase |
| Cj0581 | putative NTPase | putative NUDIX hydrolase family protein |
| Cj0594c | putative periplasmic protein | putative DNA/RNA non-specific endonuclease |
| Cj0596 | major antigenic peptide PEB3\cell binding factor 2 | major antigenic peptide PEB-cell binding factor |
| Cj0599 | putative periplasmic protein | putative OmpA family membrane protein |
| Cj0602c | hypothetical protein Cj0602c | MOSC-domain containing protein |
| Cj0604 | hypothetical protein Cj0604 | putative polyphosphate kinase |
| Cj0606 | putative periplasmic protein | putative secretion protein HlyD |
| Cj0611c | putative transmembrane transport protein | putative acyltransferase family protein |
| Cj0619 | putative integral membrane protein | putative MATE family transport protein |
| Cj0622 | transcriptional regulatory protein hypF | carbamoyltransferase |
| Cj0630c | hypothetical protein Cj0630c | putative DNA polymerase III, delta subunit |
| Cj0634 | SMF family protein | DNA processing protein A |
| Cj0635 | hypothetical protein Cj0635 | putative holliday junction resolvase |
| Cj0641 | hypothetical protein Cj0641 | putative inorganic polyphosphate/ATP-NAD kinase |
| Cj0644 | hypothetical protein Cj0644 | putative TatD-related deoxyribonuclease protein |
| Cj0647 | hypothetical protein Cj0647 | putative HAD-superfamily hydrolase |
| Cj0648 | hypothetical protein Cj0648 | putative membrane protein |
| Cj0649 | hypothetical protein Cj0649 | putative OstA family protein |
| Cj0662c | putative heat shock protein | ATP-dependent Hsl protease ATP-binding subunit |
| Cj0663c | putative heat shock protein | ATP-dependent protease HslV |
| Cj0667 | hypothetical protein Cj0667 | putative S4 domain protein |
| Cj0686 | gcpE protein homolog | 4-hydroxy-3-methylbut-2-en-1-yl diphosphate synthase |
| Cj0693c | hypothetical protein Cj0693c | S-adenosyl-methyltransferase |
| Cj0717 | hypothetical protein Cj0717 | putative ArsC family protein |
| Cj0729 | hypothetical protein Cj0729 | putative type I phosphodiesterase/nucleotide pyrophosphatase |
| Cj0733 | hypothetical protein Cj0733 | putative HAD-superfamily hydrolase |
| Cj0737 | putative periplasmic protein | putative haemagglutination activity domain containing protein |
| Cj0755 | putative iron uptake protein | ferric enterobactin uptake receptor |
| Cj0767c | 3-deoxy-D-manno-octulosonic-acid transferase | phosphopantetheine adenylyltransferase |
| Cj0769c | putative periplasmic protein | putative flagellar protein FlgA |
| Cj0770c | putative periplasmic protein | putative NLPA family lipoprotein |
| Cj0771c | putative periplasmic protein | putative NLPA family lipoprotein |
| Cj0772c | putative periplasmic protein | putative NLPA family lipoprotein |
| Cj0789 | putative RNA nucleotidyltransferase | putative multifunctional Cca protein |
| Cj0796c | hypothetical protein Cj0796c | putative hydrolase |
| Cj0800c | hypothetical protein Cj0800c | putative ATPase |
| Cj0822 | DNA /pantothenate metabolism flavoprotein | phosphopantothenoylcysteine decarboxylase |
| Cj0829c | hypothetical protein Cj0829c | putative CoA binding domain containing protein |
| Cj0832c | putative integral membrane protein | putative Na+/H+ antiporter family protein |
| Cj0841c | putative ATP/GTP binding protein | putative molybdopterin-guanine dinucleotide biosynthesis protein |
| Cj0846 | putative integral membrane protein | putative metallophosphoesterase |
| Cj0850c | transmembrane transport protein | putative MFS (Major Facilitator Superfamily) transport protein |
| Cj0883c | hypothetical protein Cj0883c | putative transcriptional regulator |
| Cj0894c | lytB homolog | 4-hydroxy-3-methylbut-2-enyl diphosphate reductase |
| Cj0917c | carbon starvation protein A homolog | putative integral membrane protein (CstA homolog) |
| Cj0921c | probable ABC-type amino-acid transporter periplasmic solute-binding protein | aspartate/glutamate-binding ABC transporter protein |
| Cj0934c | putative transmembrane transport protein | putative sodium:amino-acid symporter family protein |
| Cj0935c | putative transmembrane transport protein | putative sodium:amino-acid symporter family protein |
| Cj0941c | putative integral membrane protein | putative permease |
| Cj0943 | putative periplasmic protein | putative outer-membrane lipoprotein carrier protein precursor |
| Cj0947c | putative hydrolase | putative carbon-nitrogen hydrolase |
| Cj0948c | putative transmembrane transport protein | putative cation efflux family protein |
| Cj0949c | hypothetical protein Cj0949c | putative peptidyl-arginine deiminase family protein |
| Cj0952c | putative membrane protein | putative HAMP containing membrane protein |
| Cj0956c | putative thiophene and furan oxidation protein | putative tRNA modification GTPase |
| Cj0965c | hypothetical protein Cj0965c | putative acyl-CoA thioester hydrolase |
| Cj0976 | hypothetical protein Cj0976 | putative methyltransferase |
| Cj0981c | transmembrane transport protein | putative MFS (Major Facilitator Superfamily) transport protein |
| Cj0987c | putative integral membrane protein | putative MFS (Major Facilitator Superfamily) transport protein |
| Cj0997 | gidB homolog | putative methyltransferase (GidB homolog) |
| Cj1002c | hypothetical protein Cj1002c | putative phosphoglycerate/bisphosphoglycerate mutase |
| Cj1006c | hypothetical proteinCj1006c | putative MiaB-like tRNA modifying enzyme |
| Cj1007c | putative membrane protein | putative mechanosensitive ion channel family protein |
| Cj1011 | putative membrane protein | putative CorA-like Mg2+ transporter protein |
| Cj1013c | putative membrane protein | putative cytochrome C biogenesis protein |
| Cj1024c | signal-transduction regulatory protein | sigma-54 associated transcriptional activator |
| Cj1035c | possible transferase | putative arginyl-tRNA-protein transferase |
| Cj1039 | putative UDP-N-acetylglucosamine--N-acetylmuramyl-(pentapeptide) pyrophosphoryl-undecaprenol N-acetylglucosamine transferase | putative undecaprenyldiphospho-muramoylpentapeptide b-N-acetylglucosaminyltransferase |
| Cj1040c | putative transmembrane transport protein | putative MFS (Major Facilitator Superfamily) transport protein |
| Cj1043c | possible transferase | putative thiamine-phosphate pyrophosphorylase |
| Cj1044c | thiH protein | thiazole biosynthesis protein ThiH |
| Cj1045c | thiG protein | thiazole biosynthesis protein ThiG |
| Cj1047c | hypothetical protein Cj1047c | putative thiamine biosynthesis protein |
| Cj1049c | putative integral membrane protein | putative LysE family transporter protein |
| Cj1050c | putative transferase | NAD-dependent deacetylase |
| Cj1055c | putative integral membrane protein | putative sulfatase family protein |
| Cj1056c | hypothetical protein Cj1056c | putative carbon-nitrogen hydrolase family protein |
| Cj1060c | small hydrophobic protein | putative membrane protein |
| Cj1062 | hypothetical protein Cj1062 | putative CinA-like protein |
| Cj1068 | putative integral membrane protein | putative peptidase M50 family protein |
| Cj1080c | hypothetical protein Cj1080c | putative uroporphyrinogen-III synthase |
| Cj1087c | putative periplasmic protein | putative peptidase |
| Cj1094c | putative membrane protein | putative preprotein translocase protein |
| Cj1095 | putative integral membrane protein | putative apolipoprotein N-acyltransferase |
| Cj1104 | hypothetical proteinCj1104 | putative 4-diphosphocytidyl-2-C-methyl-D-erythritol kinase |
| Cj1107 | hypothetical protein Cj1107 | ATP-dependent Clp protease adaptor protein |
| Cj1111c | putative integral membrane protein | putative MarC family integral membrane protein |
| Cj1112c | hypothetical protein Cj1112c | putative SelR domain containing protein |
| Cj1115c | putative membrane protein | putative phosphatidylserine decarboxylase-related protein |
| Cj1117c | possible ribosomal protein methyltransferase | ribosomal protein L11 methyltransferase |
| Cj1120c | putative sugar epimerase/dehydratase | UDP-GlcNAc C4,6 dehydratase |
| Cj1121c | putative aminotransferase (degT family) | UDP-4-keto-6-deoxy-GlcNAc C4 aminotransferase |
| Cj1123c | putative transferase | acetyl transferase |
| Cj1125c | putative galactosyltransferase | GalNac transferase |
| Cj1126c | putative integral membrane protein (possible oligosaccharyl transferase) | oligosaccharide transferase |
| Cj1127c | putative glycosyltransferase | GalNac transferase |
| Cj1128c | putative glycosyltransferase | glucosyl transferase |
| Cj1129c | putative glycosyltransferase | GalNac transferase/polymerase |
| Cj1130c | ABC-type transport protein | flippase |
| Cj1131c | UDP-glucose 4-epimerase | UDP-GlcNAc/Glc 4-epimerase for LOS, capule & N-linked |
| Cj1132c | hypothetical protein Cj1132c | hypothetical protein cj1132c |
| Cj1133 | putative lipopolysaccharide heptosyltransferase | Heptosyltransferase I |
| Cj1136c | putative galactosyltransferase | putative glycosyltransferase |
| Cj1137c | hypothetical protein Cj1137c | putative glycosyltransferase |
| Cj1138 | putative galactosyltransferase | putative glycosyltransferase |
| Cj1139c | putative galactosyltransferase | Beta-1,3 galactosyltransferase addition of terminal galactose to LOS |
| Cj1140 | alpha-2,3-/alpha-2,8-sialyltransferase | alpha-2,3-sialyltransferase |
| Cj1142 | putative N-acetylglucosamine-6-phosphate 2-epimerase/N-acetylglucosamine-6-phosphatase | UDP-N-acetylglucosamine 2-epimerase |
| C1143 | acylneuraminate cytidylyltransferase | two-domain bifunctional protein (beta-1,4-N-acetylgalactosaminyltransferase/CMP-Neu5Ac synthase) |
| Cj1146 | putative glucosyltransferase | putative glycosyltransferase |
| Cj1148 | ADP-heptose--LPS heptosyltransferase | Heptosyltransferase II |
| Cj1149c | phosphoheptose isomerase 1 | sedoheptulose 7-phosphate isomerase |
| Cj1150c | putative ADP-heptose synthase | putative D-beta-D-heptose (7-phosphate kinase/1-phosphate adenylyltransferase) |
| Cj1151c | ADP-L-glycero-D-manno-heptose-6-epimerase | ADP-glyceromanno-heptose 6-epimerase |
| Cj1152c | putative phosphatase | D,D-heptose 1,7-bisphosphate phosphatase |
| Cj1154c | small hydrophobic protein | putative cytochrome oxidase maturation protein cbb3-type |
| Cj1160c | small hydrophobic protein | putative membrane protein |
| Cj1162c | hypothetical protein Cj1162c | putative heavy-metal-associated domain protein |
| Cj1170c | outer membrane protein | 50 kda outer membrane protein precursor |
| Cj1176c | hypothetical protein Cj1176c | Sec-independent protein translocase (TatA/E homolog) |
| Cj1188c | glucose inhibited division protein A homolog | tRNA uridine 5-carboxymethylaminomethyl modification enzyme |
| Cj1190c | putative MCP-domain signal transduction protein | bipartate energy taxis response protein cetA |
| Cj1191c | putative signal-transduction sensor protein | putative PAS domain containing signal-transduction sensor protein |
| Cj1198 | hypothetical protein Cj1198 | S-ribosylhomocysteine lyase (autoinducer-2 production protein LuxS) |
| Cj1200 | putative periplasmic protein | putative NLPA family lipoprotein |
| Cj1208 | hypothetical protein Cj1208 | putative 5-formyltetrahydrofolate cyclo-ligase family protein |
| Cj1209 | hypothetical protein Cj1209 | HD/KH domain containing protein |
| Cj1211 | putative integral membrane protein | putative competence family protein |
| Cj1214c | putative integral membrane protein | putative exporting protein |
| Cj1215 | putative periplasmic protein | putative peptidase M23 family protein |
| Cj1233 | putative hydrolase | putative HAD-superfamily hydrolase |
| Cj1235 | putative periplasmic protein | putative peptidase M23 family protein |
| Cj1239 | putative pyridoxal phosphate biosynthetic protein | putative 4-hydroxythreonine-4-phosphate dehydrogenase |
| Cj1241 | putative transmembrane transport protein | putative MFS (Major Facilitator Superfamily) transporter protein |
| Cj1244 | hypothetical protein Cj1244 | putative radical SAM domain protein |
| Cj1268c | hypothetical protein Cj1268c | putative FAD dependent oxidoreductase |
| Cj1270c | hypothetical protein Cj1270c | putative 2-nitropropane dioxygenase, oxidoreductase protein |
| Cj1275c | putative periplasmic protein | putative peptidase M23 family protein |
| Cj1278c | hypothetical protein Cj1278c | putative tRNA (guanine-N(7)-)-methyltransferase |
| Cj1293 | possible sugar nucleotide epimerase/dehydratase | UDP-GlcNAc-specific C4,6 dehydratase/C5 epimerase forming UDP-2-acetamido-2,6-dideoxy-β-L-arabino-hexos-4-ulose |
| Cj1294 | putative aminotransferase | C4 aminotransferase specific for PseB product |
| Cj1298 | hypothetical | putative N-acetyltransferase |
| Cj1300 | hypothetical | putative SAM domain containing methyltransferase |
| Cj1302 | hypothetical | putative HAD-superfamily phosphatase, subfamily IIIC |
| Cj1312 | possible flagellar protein | nucleotidase specific for PseC product, UDP-4-amino-4,6-dideoxy-β-L-AltNAc |
| Cj1313 | possible flagellar protein | N-acetyltransferase specific for PseC product, UDP-4-amino-4,6-dideoxy-β-L-AltNAc |
| Cj1314c | putative cyclase | imidazole glycerol phosphate synthase subunit |
| Cj1315c | amidotransferase | imidazole glycerol phosphate synthase subunit |
| Cj1316c | putative membrane protein | pseudaminic acid biosynthesis PseA protein, involved in biosynthesis or transfer of the Pse5Ac7Am |
| Cj1317 | N-acetylneuraminic acid synthetase | Pse synthetase |
| Cj1318 | hypothetical protein Cj1318 (1318 family) | (Motility accessory factor, function unknown) |
| Cj1325 | hypothetical protein Cj1325 | putative methyltransferase |
| Cj1328 | putative N-acetylglucosamine-6-phosphate 2-epimerase/N-acetylglucosamine-6-phosphatase | putative UDP-N-acetylglucosamine 2-epimerase |
| Cj1333 | hypothetical protein Cj1333 (1318 family) | PseD protein |
| Cj1334 | hypothetical protein Cj1334 (1318 family) | (Motility accessory factor, function unknown) |
| Cj1335 | hypothetical protein Cj1335 (1318 family) | (Motility accessory factor, function unknown) |
| Cj1337 | hypothetical protein Cj1337 | PseE protein |
| Cj1341c | hypothetical protein Cj1341c (1318 family) | (Motility accessory factor, function unknown) |
| Cj1342c | hypothetical protein Cj1342c (617 family) | (Motility accessory factor, function unknown) |
| Cj1368 | hypothetical protein Cj1368 | putative radical SAM domain protein |
| Cj1369 | putative transmembrane transport protein | putative permease |
| Cj1388 | hypothetical protein Cj1388 | putative endoribonuclease L-PSP |
| Cj1389 | pseudogene (transmembrane transport protein) | pseudogene (putative C4-dicarboxylate anaerobic carrier) |
| Cj1395 | pseudogene (hypothetical protein Cj1395) | pseudogene (putative MmgE/PrpD family protein) |
| Cj1397 | hypothetical protein Cj1397 | putative ferrous iron transport protein |
| Cj1404 | hypothetical protein Cj1404 | putative nicotinate-nucleotide adenylyltransferase |
| Cj1417c | hypothetical | putative amidotransferase |
| Cj1418c | hypothetical | putative transferase |
| Cj1420c | hypothetical | putative methyltransferase |
| Cj1423c | putative sugar-phosphate nucleotidyltransferase | putative D-glycero-D-manno-heptose 1-phosphate guanosyltransferase |
| Cj1425c | putative sugar kinase | putative D-glycero-D-manno-heptose 7-phosphate kinase |
| Cj1426c | hypothetical | putative methyltransferase family protein |
| Cj1428c | putative fucose synthetase | GDP-L-fucose synthase |
| Cj1430c | putative nucleotide-sugar epimerase/dehydratase | putative dTDP-4-dehydrorhamnose 3,5-epimerase |
| Cj1431c | hypothetical | capsular polysaccharide heptosyltransferase |
| Cj1435c | hypothetical | putative phosphatase |
| Cj1442c | hypothetical | putative sugar transferase |
| Cj1443c | KpsF protein | D-arabinose 5-phosphate isomerase |
| Cj1448c | putative capsule polysaccharide export system inner membrane protein | capsule polysaccharide export system inner membrane protein |
| Cj1453c | hypothetical protein Cj1453c | putative tRNA(Ile)-lysidine synthase |
| Cj1454c | hypothetical protein Cj1454c | putative radical SAM domain family protein |
| Cj1457c | hypothetical protein Cj1457c | tRNA pseudouridine synthase D |
| Cj1505c | hypothetical protein Cj1505c | putative two-component response regulator (SirA-like protein) |
| Cj1507c | hypothetical protein Cj1507c | putative regulatory protein |
| Cj1521c | hypothetical protein Cj1521c | putative CRISPR-associated protein |
| Cj1522c | hypothetical protein Cj1522c | putative CRISPR-associated protein |
| Cj1523c | hypothetical protein Cj1523c | putative CRISPR-associated protein |
| Cj1528 | pseudogene (transmembrane transport protein) | pseudogene (putative C4-dicarboxylate anaerobic carrier) |
| Cj1530 | putative ATP/GTP-binding protein | putative dephospho-CoA kinase |
| Cj1542 | hypothetical protein Cj1542 | putative allophanate hydrolase subunit 1 |
| Cj1543 | hypothetical protein Cj1543 | putative allophanate hydrolase subunit 2 |
| Cj1546 | hypothetical protein Cj1546 | putative transcriptional regulator |
| Cj1556 | hypothetical protein Cj1556 | putative transcriptional regulator |
| Cj1560 | putative membrane protein | putative permease |
| Cj1586 | putative bacterial haemoglobin | single domain haemoglobin |
| Cj1588c | putative transmembrane transport protein | putative MFS (Major Facilitator Superfamily) transport protein |
| Cj1600 | amidotransferase HisH | imidazole glycerol phosphate synthase subunit |
| Cj1603 | cyclase | imidazole glycerol phosphate synthase subunit |
| Cj1607 | hypothetical protein Cj1607 | 2-C-methyl-D-erythritol 4- phosphate cytidylyltransferase/ 2-C-methyl-D-erythritol 2,4-cyclodiphosphate synthase |
| Cj1613c | hypothetical protein Cj1613c | putative pyridoxamine 5'-phosphate oxidase |
| Cj1618c | hypothetical protein Cj1618c | putative radical SAM domain protein |
| Cj1625c | serine transporter | amino acid transporter |
| Cj1633 | hypothetical protein Cj1633 | putative ATP-binding protein |
| Cj1658 | putative integral membrane protein | putative iron permease |
| Cj1710c | hypothetical protein Cj1710c | putative metallo-beta-lactamase family protein |
| Cj1713 | hypothetical protein Cj1713 | putative radical SAM domain protein |
| Cj1724c | hypothetical protein Cj1724c | putative GTP cyclohydrolase I |

Additional file 5. CDSs modified in *C. jejuni* NCTC11168 re-annotation.
