# Supplementary material for: Re-annotation and re-analysis of the Campylobacter jejuni NCTC11168 genome sequence
Source: BMC Genomics. 2007 Jun 12;8:162. doi: 10.1186/1471-2164-8-162 (PMC1899501; doi:10.1186/1471-2164-8-162)
Supplement: Additional File 6 — Pseudogene comparison between C. jejuni NCTC11168 and C. jejuni RM1221. [file 1471-2164-8-162-S6.doc]

| **NCTC11168** | | **RM1221** | |
| --- | --- | --- | --- |
| **Gene Number** | **Product** | **Gene Number** | **Product** |
|  |  | CJE0013 | membrane protein, putative |
| Cj0046 | pseudogene (putative sodium:sulfate transmembrane transport protein) | CJE0046 | C4-dicarboxylate transporter |
| Cj0072c | pseudogene (putative iron-binding protein) | CJE0068 | conserved hypothetical protein |
|  |  | CJE0196 | citrate transporter |
| Cj0223 | pseudogene (putative IgA protease family protein) |  |  |
|  |  | CJE0274 | pathogenicity protein |
|  |  | CJE0292 | conserved hypothetical protein |
|  |  | CJE0297 | conserved hypothetical protein |
| Cj0292c | pseudogene (putative glycerol-3-phosphate transporter) | CJE0338 | glycerol-3-phosphate transporter |
| Cj0444 | pseudogene (putative TonB-denpendent outer membrane receptor) | CJE0496 | TonB-dependent receptor |
|  |  | CJE0533 | altronate hydrolase |
| Cj0501 | pseudogene (ammonium transporter) | CJE0609 | ammonium transporter |
| Cj0565 | pseudogene (conserved hypothetical protein) |  |  |
|  |  | CJE0665 | MATE efflux family protein |
|  |  | CJE0673 | conserved hypothetical protein |
|  |  | CJE0720 | hypothetical protein |
| Cj0654c | pseudogene (putative transmembrane transport protein) | CJE0758 | peptide transporter |
| Cj0676 | pseudogene (potassium-transporting ATPase A chain) | CJE0774 | potassium-transporting ATPase, A subunit |
|  |  | CJE0775 | potassium-transporting ATPase, B subunit |
| Cj0678 | pseudogene (potassium-transporting ATPase C chain) |  |  |
| Cj0742 | pseudogene (putative outer membrane protein) |  |  |
|  |  | CJE0777 | sensor histidine kinase KdpD |
|  |  | CJE0803 | conserved hypothetical protein |
|  |  | CJE0836 | conserved hypothetical protein |
| Cj0752 | pseudogene (IS element transposase) | CJE0844 | transposase |
| Cj0866 | pseudogene (arylsulfatase) | CJE0953 | arylsulfate sulfotransferase |
|  |  | CJE0955 | cytochrome c family protein |
|  |  | CJE1032 | membrane protein, putative |
|  |  | CJE1047 | conserved hypothetical protein |
|  |  | CJE1056 | conserved hypothetical protein |
|  |  | CJE1121 | DNA primase TraC |
|  |  | CJE1140 | hypothetical protein |
|  |  | CJE1175 | conserved hypothetical protein |
| Cj0969 | pseudogene (putative periplasmic protein) |  |  |
| Cj1064 | pseudogene (nitroreductase) | CJE1208 | nitroreducatase family protein |
|  |  | CJE1294 | conserved hypothetical protein |
|  |  | CJE1393 | multidrug resistance efflux transporter |
|  |  | CJE1544 | enterochelin ABC transporter |
|  |  | CJE1549 | vacuolating cytotoxin, putative |
| Cj1389 | pseudogene (putative C4-dicarboxylate anaerobic carrier) | CJE1580 | cryptic C4-dicarboxylate transporter |
| Cj1395 | pseudogene (putative MmgE/PrpD family protein) |  |  |
| Cj1470c | pesudogene (type II protein secretion system F protein) |  |  |
|  |  | CJE1585 | ferrous iron transport protein B |
|  |  | CJE1697 | CRISPR-associated protein |
| Cj1528 | pseudogene (putative C4-dicarboxylate anaerobic carrier) | CJE1699 | cryptic C4-dicarboxylate transporter |
|  |  | CJE1718 | outer membrane lipoprotein Blc |
|  |  | CJE1720 | type I restriction-modification system, R subunit |
|  |  | CJE1722 | type I restriction-modification system, S subunit |
|  |  | CJE1729 | conserved hypothetical protein |
|  |  | CJE1734 | transcriptional regulator, putative |
|  |  | CJE1759 | proline/betaine transporter, putative |
|  |  | CJE1790 | conserved hypothetical protein |
|  |  | CJE1802 | TonB-dependent receptor, putative |

Additional file 6. Comparison of pseudogenes in *C. jejuni* NCTC11168 and *C. jejuni* RM1221. Identical pseudogenes are on the same row.
